# PIM1 Promotes Survival of Cardiomyocytes by Upregulating c-Kit Protein Expression

**DOI:** 10.3390/cells9092001

**Published:** 2020-08-31

**Authors:** David E. Ebeid, Fareheh Firouzi, Carolina Y. Esquer, Julian M. Navarrete, Bingyan J. Wang, Natalie A. Gude, Mark A. Sussman

**Affiliations:** Department of Biology, San Diego State University, San Diego, CA 92182, USA; davebeid@gmail.com (D.E.E.); ffirouzi@sdsu.edu (F.F.); carolinaesquer1992@gmail.com (C.Y.E.); julianmnnavarrete@gmail.com (J.M.N.); bingyanjw@gmail.com (B.J.W.); ngude@sdsu.edu (N.A.G.)

**Keywords:** PIM1, c-Kit, cardiomyocyte, cardioprotection

## Abstract

Enhancing cardiomyocyte survival is crucial to blunt deterioration of myocardial structure and function following pathological damage. PIM1 (Proviral Insertion site in Murine leukemia virus (PIM) kinase 1) is a cardioprotective serine threonine kinase that promotes cardiomyocyte survival and antagonizes senescence through multiple concurrent molecular signaling cascades. In hematopoietic stem cells, PIM1 interacts with the receptor tyrosine kinase c-Kit upstream of the ERK (Extracellular signal-Regulated Kinase) and Akt signaling pathways involved in cell proliferation and survival. The relationship between PIM1 and c-Kit activity has not been explored in the myocardial context. This study delineates the interaction between PIM1 and c-Kit leading to enhanced protection of cardiomyocytes from stress. Elevated c-Kit expression is induced in isolated cardiomyocytes from mice with cardiac-specific overexpression of PIM1. Co-immunoprecipitation and proximity ligation assay reveal protein–protein interaction between PIM1 and c-Kit. Following treatment with Stem Cell Factor, PIM1-overexpressing cardiomyocytes display elevated ERK activity consistent with c-Kit receptor activation. Functionally, elevated c-Kit expression confers enhanced protection against oxidative stress in vitro. This study identifies the mechanistic relationship between PIM1 and c-Kit in cardiomyocytes, demonstrating another facet of cardioprotection regulated by PIM1 kinase.

## 1. Introduction

The capacity of the adult mammalian heart to regenerate following injury is severely limited [1,2,3]. The myocardial mass of the heart is 70% cardiomyocytes, the contractile units of the heart, which withdraw from the cell cycle during postnatal growth [4,5]. Pathologic injury, such as myocardial infarction (MI), causes extensive cardiomyocyte death and compromised cardiac function. In response, the heart undergoes maladaptive remodeling accompanied by biochemical, molecular, structural, and metabolic changes, placing chronic strain on cardiomyocytes and ultimately leading to heart failure [6]. Therefore, enhancing cardiomyocyte survival represents an important strategy for blunting myocardial deterioration and cardiac failure following pathological damage.

The cardioprotective role of PIM1 serine threonine kinase (Proviral Insertion site in Murine leukemia virus (PIM) kinase 1) has been extensively studied [7,8,9,10]. PIM kinases are a family of highly conserved, constitutively active serine/threonine kinases with PIM1 being the highest expressed isoform in the myocardium [9,11,12,13]. PIM1 regulates many cellular processes crucial for antagonizing cellular senescence including cell cycle progression, survival signaling, anti-apoptotic signaling, preservation of mitochondrial integrity, telomere preservation, and blunting of pathological hypertrophy [8,9,14]. Myocardial infarction injury, as represented by decreased infarct size and improved contractile performance, was mediated by cardiac specific overexpression of human PIM1 in transgenic mice [9]. Furthermore, PIM1-overexpressing hearts displayed blunting of cardiac hypertrophy, decrease in apoptosis markers, and maintenance of cardiac function following transverse aortic constriction (TAC) pressure overload compared to TAC banded non-transgenic controls [9]. In contrast, PIM1 knockout mice displayed increased cardiomyocyte apoptosis and decreased contractile function in response to MI and TAC [8,9]. Collectively, these studies demonstrate a powerful role for PIM1 in cardioprotection.

Similar to PIM1, c-Kit receptor tyrosine kinase also mediates cardioprotective activity [15]. c-Kit is expressed in diverse cardiac cell populations including endothelial cells, mesenchymal stem cells, and cardiomyocytes [16]. In response to adrenergic stress in vitro, cardiomyocytes upregulate c-Kit, implicating c-Kit in the promotion of survival signaling [17]. Functionally, the binding of c-Kit ligand, Stem Cell Factor (SCF), induces c-Kit receptor dimerization, phosphorylation, and activation of the Ras–Raf–MEK–ERK and the PI3K (Phosphoinositide 3-kinase)–Akt pathways [18,19]. The activation of ERK promotes cardiomyocyte survival in response to pressure overload, MI, and oxidative stress [20,21,22]. The activation of Akt promotes phosphorylation and inactivation of pro-apoptotic proteins [23]. Taken together, these results point to a cardioprotective cascade induced by c-Kit activity.

PIM1 regulates c-Kit protein levels in hematopoietic stem cells, altering cellular biological properties including colony-forming capacity [24]. The present study was designed to delineate the relationship between PIM1 and c-Kit in cardiomyocytes to reveal a cardioprotective signaling cascade contributing to the amelioration of cardiomyopathic injury. The findings from this study demonstrate that PIM1 upregulates c-Kit expression post-transcriptionally in cardiomyocytes. PIM1 interacts with c-Kit at the protein level offering a potential mechanism of PIM1-mediated regulation of c-Kit expression. Furthermore, cardiomyocytes with elevated c-Kit expression demonstrate enhanced resistance to oxidative stress in vitro. Collectively, the findings of this study establish a novel signaling relationship for PIM1/c-Kit-mediated cardiomyocyte survival.

## 2. Materials and Methods

### 2.1. Animal Experiments

All animal protocols were approved by the Institutional Animal Care and Use Committee (IACUC) of San Diego State University (APF# 19-03-003S, approval date 9 May 2019) and conform to the Guide for the Care and Use of Laboratory Animals published by the US National Institutes of Health. Transgenic mice with cardiac specific overexpression of human PIM1 (PIM1) and global deletion of PIM1, PIM2, and PIM3 triple knockout (PIM-TKO) have been described before [9,25]. Non-transgenic (NTg) and gender matched mice of the same strain (FVB) were used as controls. Adult mice aged 4–6 months were used for the study.

### 2.2. Isolation and Culture of Adult Mouse Cardiomyocytes

Cardiomyocytes were isolated from FVB mice as previously described [26]. Briefly, mice were anesthetized with a xylazine/ketamine solution and injected with Heparin (100 U/kg) (Sigma-Aldrich; St. Louis, MO, USA; H3393) intraperitoneally to prevent blood clots. The chest was opened, and the aortic arch was isolated. Curved forceps were used to push a 4-0 suture underneath the aorta. A small incision was made at the aortic branch and a cannula was inserted into the aorta then tied securely with the suture. The cannula was attached to a Langendorff apparatus and was perfused until all blood was cleared from the heart. The heart was then digested with using buffer containing Collagenase II (230 U/mL) (Worthington; Columbus, OH, USA; LS004147) for 12 min. Atria were removed, left and right ventricles were gently teased apart using forceps and triturated until no large tissue remained. The cell suspension was transferred to a T-75 flask and calcium was gradually introduced over a 30-min interval to a final concentration of 900 µm. Following calcium introduction, cardiomyocytes were either lysed for protein or were pre-plated on laminin (ThermoFisher; Waltham, MA, USA; 23017015)-coated dishes at a density of 50,000 cells/mL. After 2 h, cardiomyocytes were cultured in serum-free media overnight at 37 °C, following which pharmacological treatments or cell death experiments were conducted.

### 2.3. Viral Infections

Cardiomyocytes were infected with adenovirus encoding enhanced green fluorescent protein (eGFP) or mouse c-Kit-GFP (VectorBuilder; Chicago, IL, USA; VB190910-1241cgg) at the multiplicity of infection (MOI) of 100 for 48 h. GFP and c-Kit overexpression were confirmed via immunoblot.

### 2.4. Pharmacological Treatments

Cardiomyocytes were plated at a density of 100,000 cells per 35 mm dish. The next day, cardiomyocytes were treated with 200 ng/mL murine stem cell factor (SCF) (Peprotech; Rocky Hill, NJ, USA; 250-03) for one hour. For the cell death assay, cardiomyocytes were pre-treated with 0.5 µM Imatinib (R&D Systems; Minneapolis, MN, USA; 5906) for 2 h.

### 2.5. Immunoblot

Cells were lysed in ice cold radioimmunoprecipitation assay (RIPA) buffer (ThermoFisher; Waltham, MA, USA; 89900) containing protease and phosphatase inhibitor cocktails (Sigma-Aldrich; P8340, P5726, P2850). Protein concentrations were analyzed and normalized by Bradford Assay and lysates were prepared by addition of NuPAGE LDS Sample Buffer (ThermoFisher; NP0007) and 100 µM dithiothreitol (Bio-Rad; 161-0611). Samples were sonicated and boiled then loaded onto a 4–12% NuPAGE Bis-Tris gel (ThermoFisher; NP0321BOX) and run in MOPS buffer (ThermoFisher; NP0001) for 80 min at 150 V. Proteins were transferred onto an Immobilon-FL Polyvinylidene difluoride membrane (EMD Millipore; Burlington, MA, USA; IPFL0010) for 2 h at 30 mA, following which the membrane was blocked with Odyssey Blocking Buffer (Li-Cor; Lincoln, NE, USA; 927-50000) for one hour at room temperature and incubated with primary antibodies prepared in blocking buffer with 0.2% Tween-20 overnight at 4 °C. Secondary antibodies prepared in blocking buffer were applied for 2 h at room temperature. Fluorescent signal was detected using an Odyssey CLx imaging system (Li-Cor) and bands were quantified using Image Studio. The primary antibodies used are listed in Table 1.

### 2.6. Quantitative RT-PCR

RNA was isolated using the Quick-RNA Miniprep Kit (Zymo Research; Irvine, CA, USA; R1054) following the manufacturer’s instructions. RNA concentrations were determined using a Nanodrop 2000 spectrophotometer (ThermoFisher) and cDNA was synthesized using the iScript cDNA synthesis kit (Bio-Rad; Hercules, CA, USA; 1708890). Reactions were prepared in triplicate using 6.5 ng cDNA per reaction and iQ SYBER Green (Bio-Rad; 1708880) on a CFX Real-Time PCR thermocycler (Bio-Rad). Samples were normalized to 18S and data were analyzed by the ∆∆Ct method. The primer sequences used are as follows:

c-Kit Forward: ATTGTGCTGGATGGATGGAT
c-Kit Reverse: GATCTGCTCTGCGTCCTGTT
Co-Immunoprecipitation

PIM1 hearts were transferred to a 5 mL round bottom tube containing 1 mL of lysis buffer composed of Tris-HCl, pH 7.4 (50 mm), NaCl (150 mm), Ethylenediaminetetraacetic acid (EDTA, 1 mm), Egtazic acid (EGTA, 1 mm) and protease and phosphatase inhibitors (Sigma-Aldrich). The hearts were finely minced using a Polytron Homogenizer (ThomasSci; Swedesboro, NJ, USA) at 22,000 RPM (Revolutions Per Minute) for 3 s. The homogenates were transferred to a 1.7 mL microcentrifuge tube and Triton X-100 was added to a final concentration of 1%. The tissue homogenates were vortexed then incubated on ice for 45 min. Debris was cleared from the homogenates by centrifugation at 20,000× *g* at 4 °C. The lysates were precleared by addition of 20 µL Protein A agarose beads (Santa Cruz Biotechnology; Dallas, TX, USA; sc-2001) on a rotator at 4 °C for 30 min. The beads were pelleted by centrifugation at 1000× *g* for 5 min and the supernatant was transferred to a fresh microcentrifuge tube on ice. An aliquot of the sample was taken as the “pre-immunoprecipitation” sample. Primary antibody was added to the lysates and was incubated on a rotator at 4 °C overnight. Antibodies are listed in Table 1. The following day, 20 µL Protein A agarose beads were added to the lysates on a rotator for 2 h. The beads were pelleted by centrifugation at 1000× *g* for 5 min and the supernatant was saved as the “post-immunoprecipitation supernatant”. The beads were washed by resuspending in 1 mL of ice-cold phosphate-buffered saline (PBS), following which the beads were centrifuged. The washes were repeated for a total of 3 times. The bead pellet was suspended in NUPAGE LDS sample buffer (ThermoFisher; NP0007) containing 100 µM dithiothreitol (Bio-Rad; 161-0611) and the beads were boiled at 95 °C for 5 min. The sample was centrifuged at 20,000× *g* at room temperature for 5 min and the samples were loaded on a gel for immunoblot analysis.

### 2.7. Proximity Ligation Assay

Cardiomyocytes were isolated and plated on 8 well glass chamber slides. Cells were fixed in a 4% formaldehyde solution for 20 min at room temperature then were permeabilized in a 0.1% Triton-X solution for 15 min. The proximity ligation assay was performed according to the manufacturer’s instructions (Sigma Aldrich; DUO92104-1KT). Briefly, cardiomyocytes were blocked using Duolink Blocking Solution in a heated humidity chamber for 60 min at 37 °C. Primary antibody was diluted in Duolink Antibody Diluent and added to the wells. The slides were incubated with primary antibody overnight in a humidity chamber at 4 °C. The following day, the slides were washed twice for 5 min each using Wash Buffer A. The plus and minus probes were applied to the wells and the slides were incubated in a humidity chamber for 60 min at 37 °C. The slides were washed twice for 5 min each using Wash Buffer A, following which the ligation solution was added and incubated for 30 min at 37 °C. After two 5-min washes using Wash Buffer A, the amplification solution was added to the wells and the slides were incubated for 100 min at 37 °C. The slides were washed twice for 10 min each using Wash Buffer B, followed by one last wash in 0.01× Wash Buffer B for 1 min. The slides were then mounted with a coverslip using Mounting Medium with 4′,6-diamidino-2-phenylindole (DAPI) and were imaged using a Leica SP8 confocal microscope.

### 2.8. Cell Death Assay

Cardiomyocytes were isolated and plated on 35 mm dishes. Following viral or pharmacological treatment, cells were treated with 10 µm H_2_O_2_ (Sigma Aldrich; H1009) for 3 h. TO-PRO-3 (ThermoFisher; T3605) was added to the wells in a 1:10,000 dilution and cells were immediately imaged using a Leica SP8 confocal microscope.

### 2.9. Statistical Analyses

Statistical analyses were performed using a Student’s t-test, one way-ANOVA or two-way ANOVA with Tukey’s multiple comparison test on GraphPad Prism v5.0. A value of *p* < 0.05 was considered statistically significant.

## 3. Results

### 3.1. PIM1 Upregulates c-Kit Protein Expression

**Figure 1:**
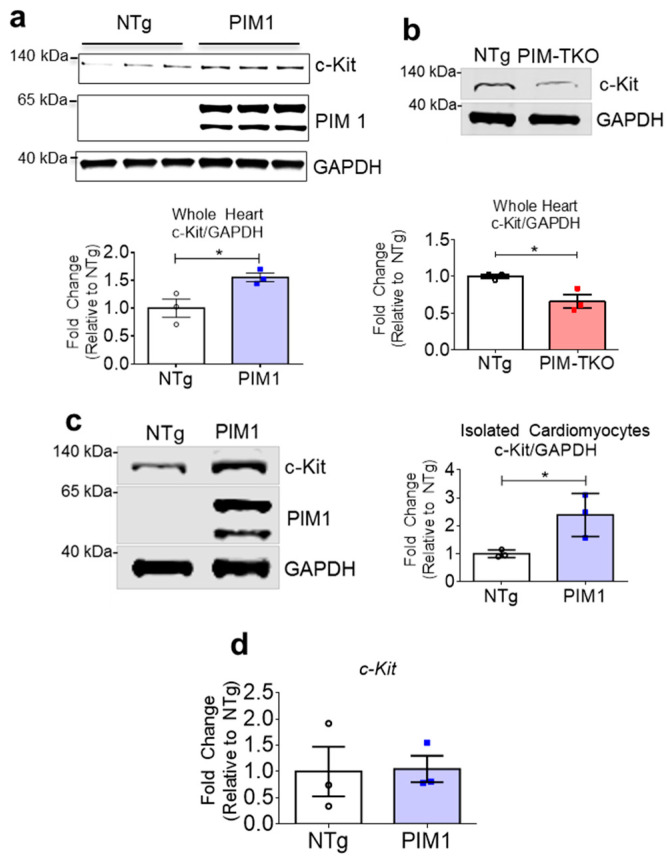
PIM1 upregulates c-Kit protein expression. (**a**) Immunoblot analysis showing expression of c-Kit in PIM1 vs. NTg whole heart lysates with quantification below. N = 3, Error bars represent SEM, * *p* < 0.05 vs. untreated as measured by Student t test. (**b**) Immunoblot analysis showing the expression of c-Kit in PIM-TKO vs. NTg whole heart lysates with quantification shown below. N = 3, error bars represent SEM, * *p* < 0.05 vs. untreated as measured by Student t test. (**c**) Immunoblot analysis showing c-Kit expression in cardiomyocytes isolated from PIM1-overexpressing hearts vs. NTg with quantification on the right. N = 3, error bars represent SEM, * *p* < 0.05 vs. untreated as measured by Student t test. (**d**) Gene expression of c-Kit in cardiomyocytes isolated from PIM1 and NTg hearts as revealed by qPCR. N = 3, Error bars represent SEM.

PIM1 regulates c-Kit expression in hematopoietic stem cells [24], but this relationship has not been examined in the myocardial context. Therefore, c-Kit protein level was measured in whole heart lysates from PIM1 and PIM-triple knockout (TKO) mice by immunoblot. PIM1 overexpression is driven downstream of the alpha myosin heavy chain promotor, which results in cardiomyocyte-specific overexpression of PIM1. PIM1 hearts displayed significantly elevated c-Kit expression compared to non-transgenic (NTg) hearts (Figure 1a and Figure S1a), whereas PIM-TKO hearts displayed significantly lower c-Kit expression compared to NTg (Figure 1b and Figure S1b). To further validate the role of PIM1 in the regulation of c-Kit expression, cardiomyocytes were isolated and analyzed for c-Kit expression via immunoblot. PIM1 cardiomyocytes displayed a 2.5-fold increase in c-Kit expression compared to NTg cardiomyocytes (Figure 1c and Figure S1c). The c-Kit mRNA level was analyzed by RT-qPCR, revealing no significant difference between PIM1 and NTg cardiomyocytes, consistent with the premise that PIM1 does not regulate c-Kit at the transcriptional level (Figure S1d). Overall, these results demonstrate that PIM1 regulates c-Kit expression post-transcriptionally in adult murine cardiomyocytes.

### 3.2. PIM1 Interacts with c-Kit

**Figure 2:**
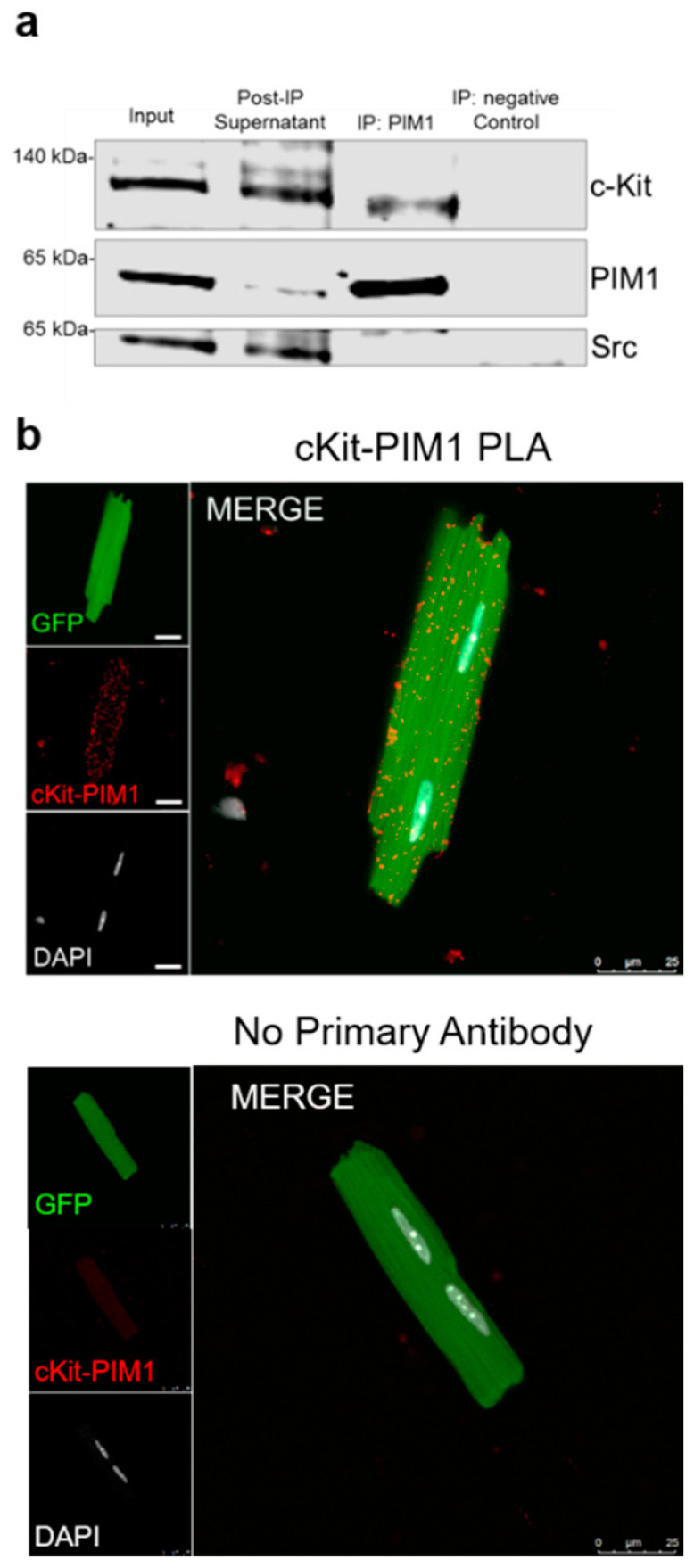
PIM1 interacts with c-Kit. (**a**) Immunoblot analysis of a co-immunoprecipitation using whole heart lysates from PIM1 hearts in which PIM1 was pulled down. (**b**) Interaction between PIM1 and c-Kit as determined by proximity ligation assay (top panel) and in the absence of primary antibody (bottom panel). Proximity events are shown as red dots.

Regulation of c-Kit expression by PIM1 occurs post-transcriptionally ([24], Figure 1), therefore the potential association between PIM1 and c-Kit proteins in heart lysates was assessed by co-immunoprecipitation (IP) of c-Kit and PIM1 followed by immunoblotting (Figure 2a and Figure S2). PIM1-overexpressing whole heart input lysates displayed elevated expression of PIM1, c-Kit, and SRC. Levels of PIM1 were decreased in post-IP supernatant following pull down with PIM1 antibody, validating the depletion of PIM1 from heart lysate. PIM1 and c-Kit were enriched in the IP: PIM1 sample, as evidenced by the prominent bands corresponding to PIM1 protein and c-Kit (Figure 2a), confirming a protein association between PIM1 and c-Kit. No c-Kit was detected in the negative control, indicating that c-Kit was only enriched in association with PIM1 IP. Protein expression of SRC, a tyrosine kinase that has no known interaction with PIM1, was also analyzed as a negative-interaction protein control. PIM1 IP failed to show an association with the SRC protein, supporting the specificity of the PIM1:c-Kit interaction. For further corroboration of the PIM1:c-Kit association, a proximity ligation assay (PLA) was performed on isolated PIM1 cardiomyocytes. PLA demonstrates a physical association and protein–protein interaction of two separate molecules within 40 nm (Figure 2b). Proximity between PIM1 and c-Kit was detected in cardiomyocytes, as indicated by the red fluorescence, which displayed a significantly higher number of spots of proximity compared to the negative controls (Figure S3). Taken together, these results demonstrate that PIM1 interacts with c-Kit at the protein level in cardiomyocytes.

### 3.3. Cardioprotective Signaling Downstream of c-Kit is Elevated in PIM1 Cardiomyocytes

**Figure 3:**
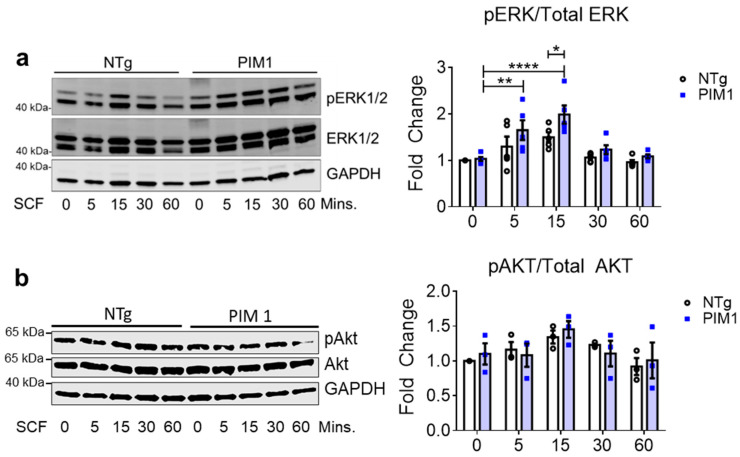
Cardioprotective signaling downstream of c-Kit is elevated in PIM1 cardiomyocytes. (**a**) Immunoblot analysis of activated ERK1/2 and (**b**) activated AKT in NTg and PIM1 cardiomyocytes following treatment with SCF over 60 min. Quantification is shown on the right. N = 5, Error bars represent SEM, * *p* < 0.05, ** *p* < 0.01 and **** *p* < 0.0001 as measured by two-way ANOVA multiple comparison with Tukey.

c-Kit receptor activation is mediated by the binding of Stem Cell Factor (SCF) that activates the cardioprotective PI3k-Akt and MAPK/ERK pathways [20,22,23,27]. Signal cascades downstream of c-Kit receptor activation by SCF were assessed to determine the cardioprotective signaling upregulation in PIM1 versus normal cardiomyocytes. Isolated cardiomyocytes from PIM1-overexpressing and non-transgenic (NTg) hearts were treated with SCF and levels of activated ERK1/2 and AKT were measured over a one-hour time course by immunoblot. Cardiomyocytes from both groups responded to SCF treatment with the activation of AKT or ERK signaling (Figure 3) unaffected by the overexpression of c-Kit (Figure 1c). In contrast, PIM1 cardiomyocytes displayed significantly increased ERK1/2 activation at the 15-min time point compared to NTg cardiomyocytes (Figure 3 and Figure S4a). AKT was not significantly different between PIM1 and NTg cardiomyocytes over the time course (Figure 3b and Figure S4b). In summary, PIM1 cardiomyocytes responded to SCF treatment by activating both ERK and AKT, but only ERK1/2 activation was enhanced by the presence of PIM1.

### 3.4. Elevated c-Kit Expression Enhances Resistance Against Oxidative Stress in Cardiomyocytes

**Figure 4:**
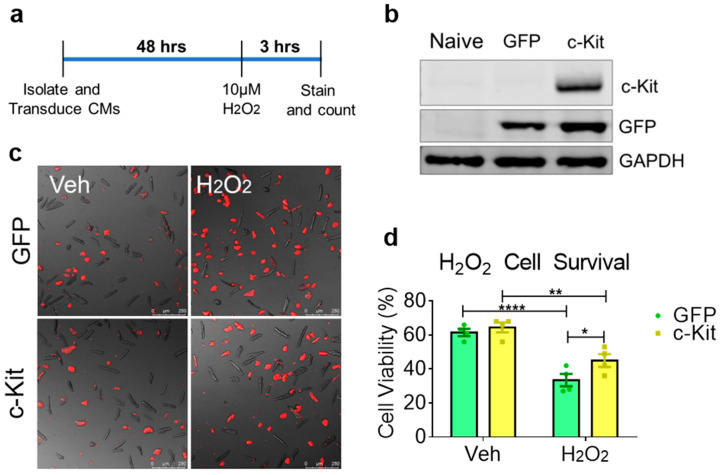
Cardiomyocytes with elevated c-Kit expression demonstrate enhanced resistance against oxidative stress. (**a**) Schematic showing the treatment procedure. (**b**) Immunoblot analysis of isolated cardiomyocytes transduced with GFP or c-Kit-GFP. (**c**) Fluorescent images of TO-PRO-3 staining on GFP or c-Kit-expressing cardiomyocytes treated with vehicle or H_2_O_2_ overlaid with brightfield images depicting dead or dying cells in red. (**d**) Quantification of cell viability counted from fluorescent images. N = 4, 300–600 cardiomyocytes counted per group, error bars represent SEM, * *p* < 0.05, ** *p* < 0.01 and **** *p* < 0.0001 as measured by two-way ANOVA multiple comparison with Tukey.

c-Kit activity is cardioprotective [28], raising the possibility that elevated c-Kit expression enhances the survival of cardiomyocytes. The elevation of c-Kit protein was mediated by adenoviral overexpression in cardiomyocytes (Figure 4a). Validation of adenoviral expression of GFP and c-Kit was confirmed via immunoblot (Figure 4b and Figure S5). c-Kit overexpression sufficiently activated cardioprotective signaling in cardiomyocytes, as evidenced by the upregulation of phosphorylated ERK1/2 and AKT (Figure S6). After viral transduction to induce protein overexpression and activation of downstream signaling, the cardiomyocytes were treated with H_2_O_2_ and cell death was determined using TO-PRO-3 (Figure 4c). Cell viability was comparable between c-Kit-overexpressing cardiomyocytes compared to the GFP controls at baseline (Figure 4d). Viability was significantly higher in c-Kit-overexpressing cardiomyocytes following H_2_O_2_-induced stress compared to the GFP cardiomyocytes, although both groups showed comparable live cells at baseline prior to exposure (Figure 4c,d—Vehicle only). Collectively, these results demonstrate that elevated c-Kit expression protects cardiomyocytes against oxidative stress.

### 3.5. PIM1 Confers Cardioprotection Independent of c-kit Activity 

**Figure 5:**
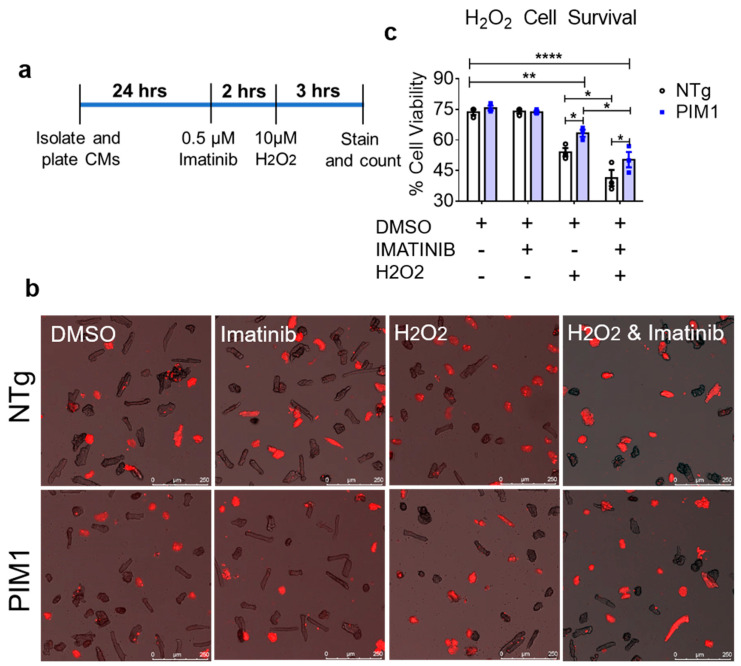
PIM1 and c-Kit act independently to confer cardioprotection. (**a**) schematic showing the treatment procedure. (**b**) Representative images of NTg and PIM1 cardiomyocytes stained with TO-PRO-3. Cells depicted in red are counted as non-viable cells. (**c**) Quantification of cell viability counted from fluorescent images. N = 5, 300–600 cardiomyocytes counted per group, error bars represent SEM, * *p* < 0.05, ** *p* < 0.01 and **** *p* < 0.0001 as measured by two-way ANOVA multiple comparison with Tukey.

The cardioprotective role of PIM1 is well established [8,9,10,29,30,31] but the involvement of c-Kit in PIM1-mediated cardioprotection has not been elucidated. The involvement of c-Kit in PIM1-mediated cardioprotection was assessed in cardiomyocytes subjected to oxidative stress following treatment with imatinib, an inhibitor of c-Kit activity [32]. Isolated cardiomyocytes were pretreated with imatinib for 2 h, then challenged with H_2_O_2_ for 3 h (Figure 5a). The inhibition of tyrosine kinase activity with Imatinib blunted AKT activation in NTg but not PIM1-overexpressing cardiomyocytes. PIM1-overexpressing cells exhibited significantly enhanced AKT activation compared to the NTg cells upon Imatinib treatment. ERK1/2 activation was similarly impacted by Imatinib in NTg cardiomyocytes albeit at a modest level, resulting in a higher level of ERK1/2 activation in PIM1-overexpressing cells in response to oxidative stress (Figure S7). Cell viability was determined by quantitation of TO-PRO-3-stained cardiomyocytes (Figure 5b). Viability was comparable between PIM1 and NTg cardiomyocytes at baseline prior to H_2_O_2_-mediated stress (Figure 5c) and imatinib treatment had no effect on cell viability. In comparison, viability was significantly higher following H_2_O_2_ exposure in PIM1 cardiomyocytes compared to NTg cells. Pretreatment with imatinib significantly decreased cell viability following the H_2_O_2_ challenge in PIM1 and NTg cardiomyocytes compared to H_2_O_2_ alone. However, PIM1 cardiomyocytes retained higher cell viability compared to NTg even after imatinib-blocking of receptor tyrosine kinase activity. Taken together, these results are consistent with PIM1 acting both independently and synergistically with c-Kit to protect cardiomyocytes from oxidative stress.

## 4. Discussion

Decades of cardioprotective signal transduction studies have produced an intricate web of relationships between mediators of cardiomyocyte survival. Our group were pioneers in establishing PIM1-mediated cardioprotection in over a decade of studies [8,9,16,29,30,33,34,35], which was subsequently reinforced by studies from other researchers [7,10,36,37]. Similarly, cardioprotection is also conferred by c-Kit activity [17,32,38], with c-Kit biology inextricably linked to myocardial homeostasis [39,40]. Synergism between PIM1 and c-Kit in cardioprotective signaling could be inferred from fundamental biological roles in hematopoietic cells for both PIM1 [11,41,42] and c-Kit [6,43] as well as the ability of PIM1 to increase c-Kit gene translation [24]. Following these precedents, this study establishes the link between c-Kit and PIM1 in cardiomyocyte protection from oxidative stress in vitro. Specifically, PIM1 interacts with c-Kit protein to increase c-Kit expression, which subsequently promotes the activation of ERK but not AKT, consistent with the distinct involvement of the AKT and ERK1/2 signaling pathways to mediate c-Kit functions in cardiomyocytes [44,45,46,47,48,49]. Although PIM1 and c-Kit could act synergistically to confer cardioprotection, PIM1-mediated cardioprotective signaling in cardiomyocyte is partially independent of c-Kit. Imatinib inhibits tyrosine kinase activity not only through c-Kit but also potentially through abl (the Abelson proto-oncogene) and PDGF-R (platelet-derived growth factor receptor). Even after Imatinib-mediated inhibition of tyrosine kinase activity, PIM1-overexpressing cardiomyocytes exhibit enhanced ERK1/2 and AKT activation and, subsequently, higher survival rates. These findings provide novel insights into the PIM1-mediated signal transduction mechanisms of cardioprotection.

Collectively, the present study, along with previously published reports, reinforces the crucial role of PIM1 in promoting cardioprotective signaling. The overexpression of PIM1 in neonatal rat cardiomyocytes (NRCM) upregulated anti-apoptotic proteins BCL-XL and BCL-2 and inactivated pro-apoptotic protein BAD by phosphorylation [9]. Further, PIM1 overexpression protected mitochondrial integrity and decreased mitochondrial swelling in NRCM after hydrogen peroxide treatment [14]. In vivo studies also demonstrated the cardioprotective role of PIM1. PIM1 knockout mice displayed decreased contractile function and increased apoptotic death in response to MI and TAC [8,9]. Conversely, mice with cardiac-specific overexpression of PIM1 displayed decreased apoptotic cell death after MI, blunting of hypertrophy, decreased infarct size after MI, and preserved contractility [9].

Elevated levels of c-Kit enhance resistance to oxidative stress in vitro by promoting the downstream expression of activated ERK. The Ras–Raf–MEK–ERK cascade has been implicated in promoting the survival of cardiomyocytes in response to oxidative stress [17,19,21,50,51].

While the present study introduces a link between PIM1 and c-Kit in cardiomyocytes, the mechanism of PIM1-mediated upregulation of c-Kit remains to be elucidated. A previous report demonstrating PIM1 regulation of c-Kit in hematopoietic cells found regulation to be at the translational level [24]. Knockout of PIM1 did not affect transcription of the c-Kit gene, but PIM1 expression enhanced c-Kit 35S methionine labeling and increased the ribosomal incorporation of c-Kit mRNA [24]. Possibilities for how PIM1 exerts potentiation of c-Kit expression could include direct protein–protein interaction, as well as post-translational modifications and stabilization of c-Kit, analogous to the effect of PIM1 upon RelA/P65 phosphorylation [52]. The potential of PIM1 to phosphorylate c-Kit is one of several possibilities based upon our findings to be investigated in the future.

In summary, PIM1 upregulates c-Kit protein expression to promote protection in cardiomyocytes, consistent with cumulative evidence of cardioprotection as well as preservation of a more “youthful” phenotype mediated by PIM1 overexpression [9,29,34,53]. Reinforcement of c-Kit activity by PIM1 could account for the ability of PIM1 to antagonize cellular senescence [33,53] and serve as the basis for future studies. Mechanistic understanding of PIM1-mediated cardioprotection could provide valuable information towards the protection of cardiomyocytes to promote cardiac repair.

## Figures and Tables

**Table 1:** Antibody list.

Antibody	Catalog Number	Dilution	Application
PIM1	ThermoFisher 39-4600	1:500	IB, Co-IP
PIM1	ThermoFisher 710504	1:100	PLA
c-Kit	R&D AF1356	1:200 1:100	IB, PLA
GAPDH (Glyceraldehyde 3-phosphate) dehydrogenase	Millipore Sigma MAB374	1:5000	IB
SRC	Abcam Ab47405	1:500	IB
pERK1/2 Phosphorylated ERK1/2	CST 9101	1:500	IB
ERK1/2	CST 9102	1:500	IB
pAkt (Ser473) Phosphorylated Akt at serine 473	CST 9271	1:500	IB
Akt	CST 9272	1:500	IB
GFP (Green Fluorescent Protein)	Rockland 600-101-215	1:1000	IB

## Supplementary Materials

The following are available online at https://www.mdpi.com/2073-4409/9/9/2001/s1, Figure S1: (a) Full blot of cropped image presented in Figure 1a, (b) Figure 1b, and (c) Figure 1c of the manuscript. (d) Gene expression of c-Kit in cardiomyocytes isolated from PIM1 and NTg hearts as revealed by qPCR. N = 3, error bars represent SEM, Figure S2: Full blot of cropped image presented in Figure 2a of the manuscript, Figure S3: Negative controls for the Proximity Ligation Assay. Endogenous GFP is shown in green, the PLA signal is shown in red and DAPI is shown in gray, Figure S4: Full blot of cropped image presented in Figure 3a,b of the manuscript, Figure S5: Full blot of cropped image presented in Figure 4b of the manuscript, Figure S6: Immunoblot analysis of c-Kit, activated ERK1/2 and activated AKT in naïve and virally transduced cardiomyocytes with quantification shown below. Error bars represent SEM, * *p* < 0.05, ** *p* < 0.01 and *** *p* < 0.001 as measured by two-way ANOVA, multiple comparison with Tukey, Figure S7: Immunoblot analysis of activated ERK1/2 and activated AKT in NTg and PIM1 overexpressing cardiomyocytes in response to oxidative stress in presence and absence of Imatinib. Quantification is shown below. Error bars represent SEM, ** *p* < 0.01 as measured by two-way ANOVA, multiple comparison with Tukey.
