# Supplementary material for: PIM1 Promotes Survival of Cardiomyocytes by Upregulating c-Kit Protein Expression
Source: Cells. 2020 Aug 31;9(9):2001. doi: 10.3390/cells9092001 (PMC7563506; doi:10.3390/cells9092001)
Supplement: Supplementary file 1 [file cells-09-02001-s001.pdf]

# PIM1 Promotes Survival of Cardiomyocytes by Upregulating c-Kit Protein Expression

*Cells*

David E Ebeid [1]*, Fareheh Firouzi[1]*, Carolina Y Esquer[1], Julian M Navarrete[1], Bingyan J Wang[1], Natalie A Gude[1], Mark A Sussman[1#]

1 Department of Biology, San Diego State University, San Diego, CA, USA

*** D.E. and F.F. contributed equally to this manuscript.**
# Correspondence:
Mark A. Sussman, PhD
(619)-594-2983
heartman4ever@icloud.com

# Supplemental Figure 1

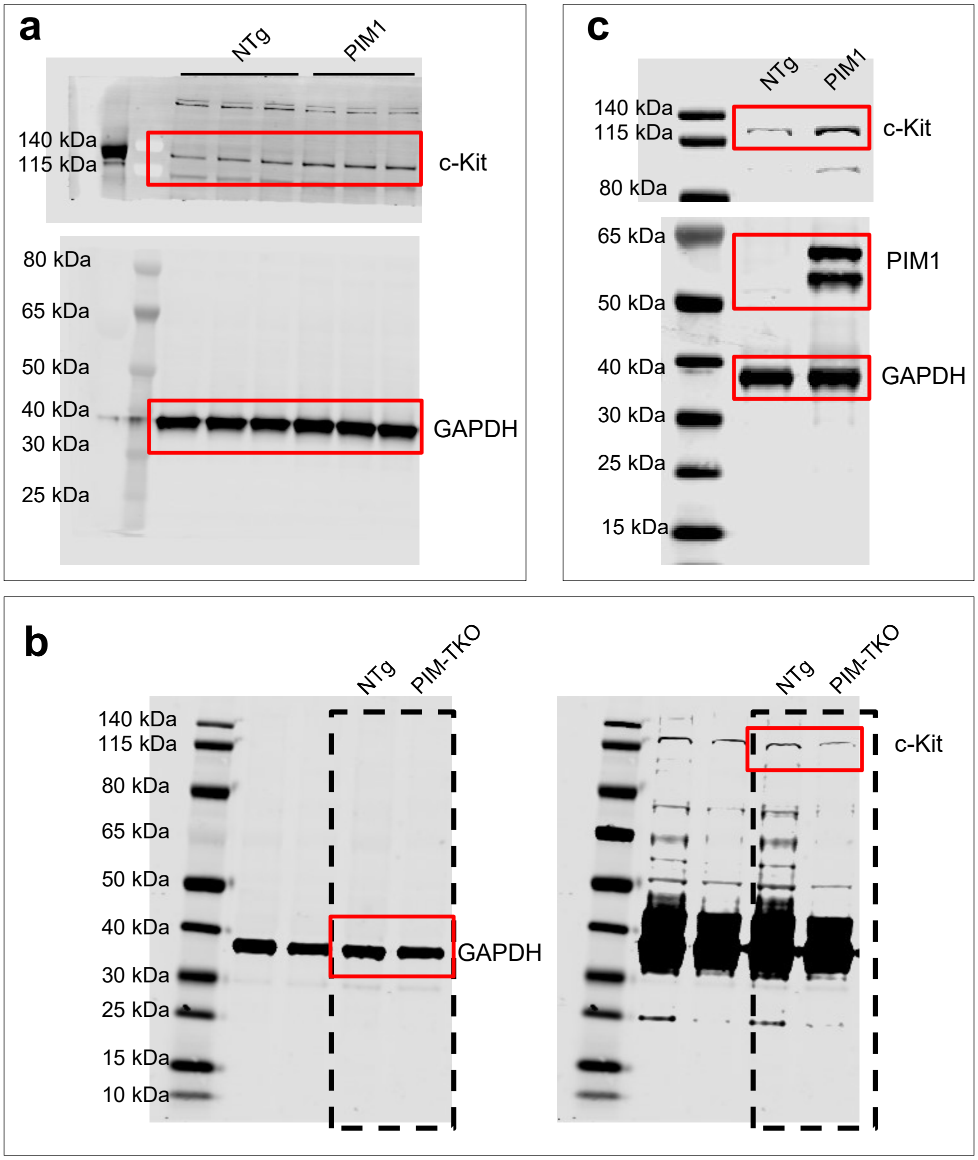


**Fig S1**: **a** Full blot of cropped image presented in Fig 1a, **b** Fig 1b, and **c** Fig 1c of the manuscript.

# Supplemental Figure 2

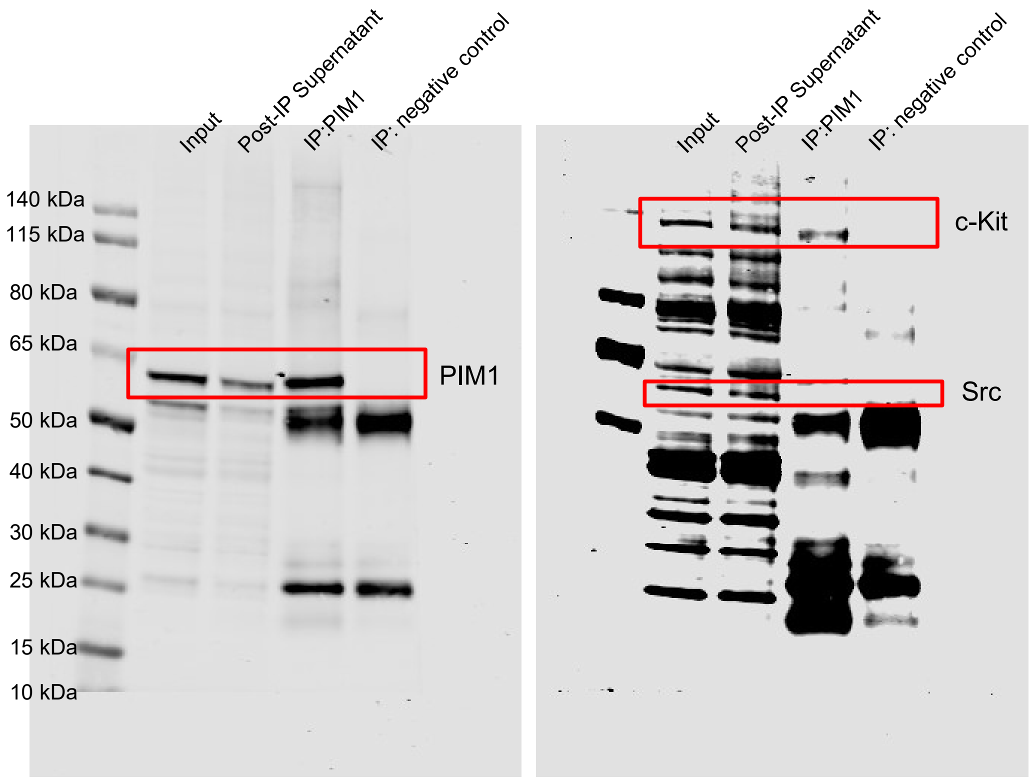


**Fig S2**: Full blot of cropped image presented in Fig 2a of the manuscript.

# Supplemental Figure 3

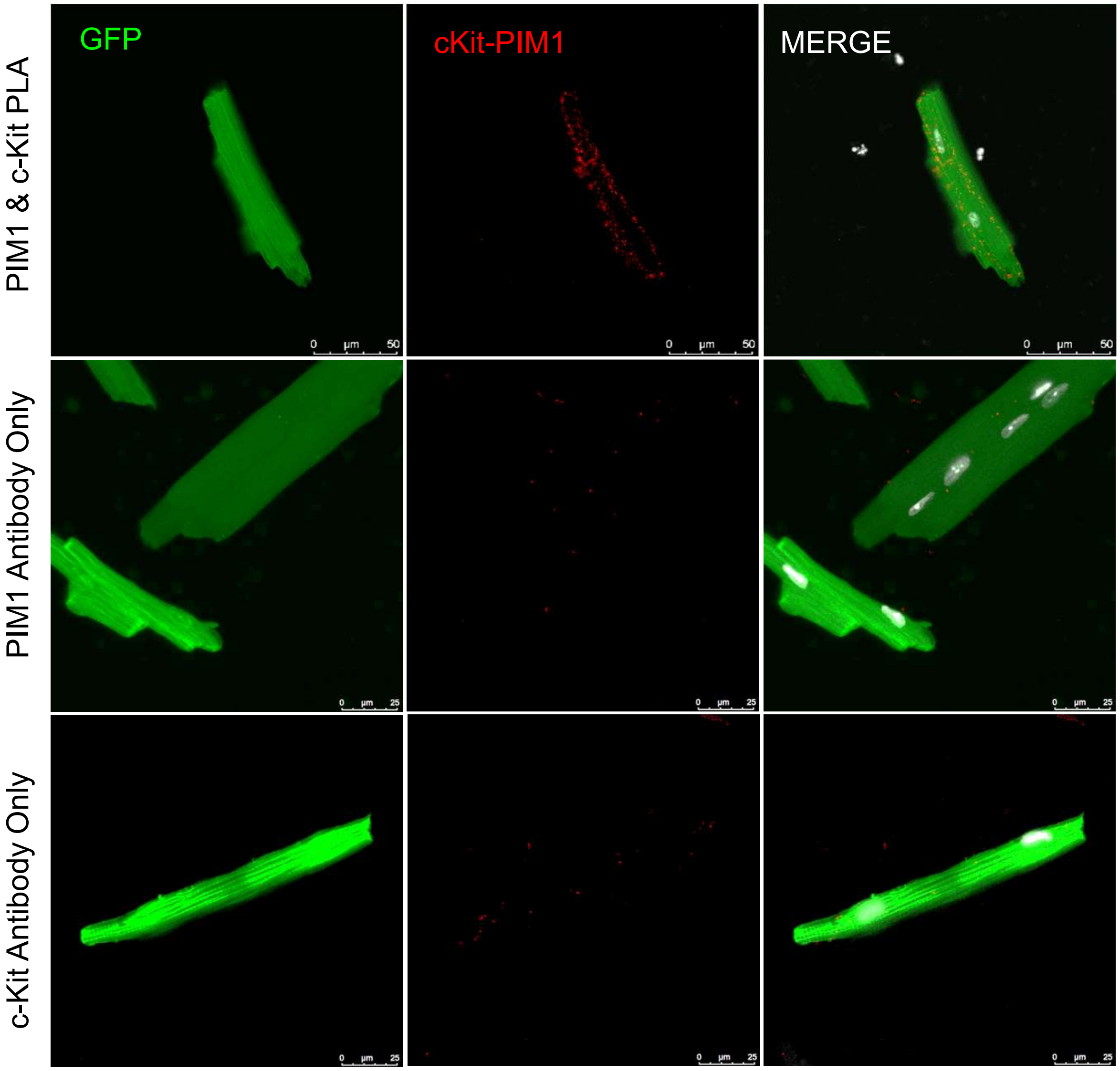


**Fig S3**: Negative controls for the Proximity Ligation Assay. Endogenous GFP is shown in green, the PLA signal is shown in red and DAPI is shown in gray.

# Supplemental Figure 4

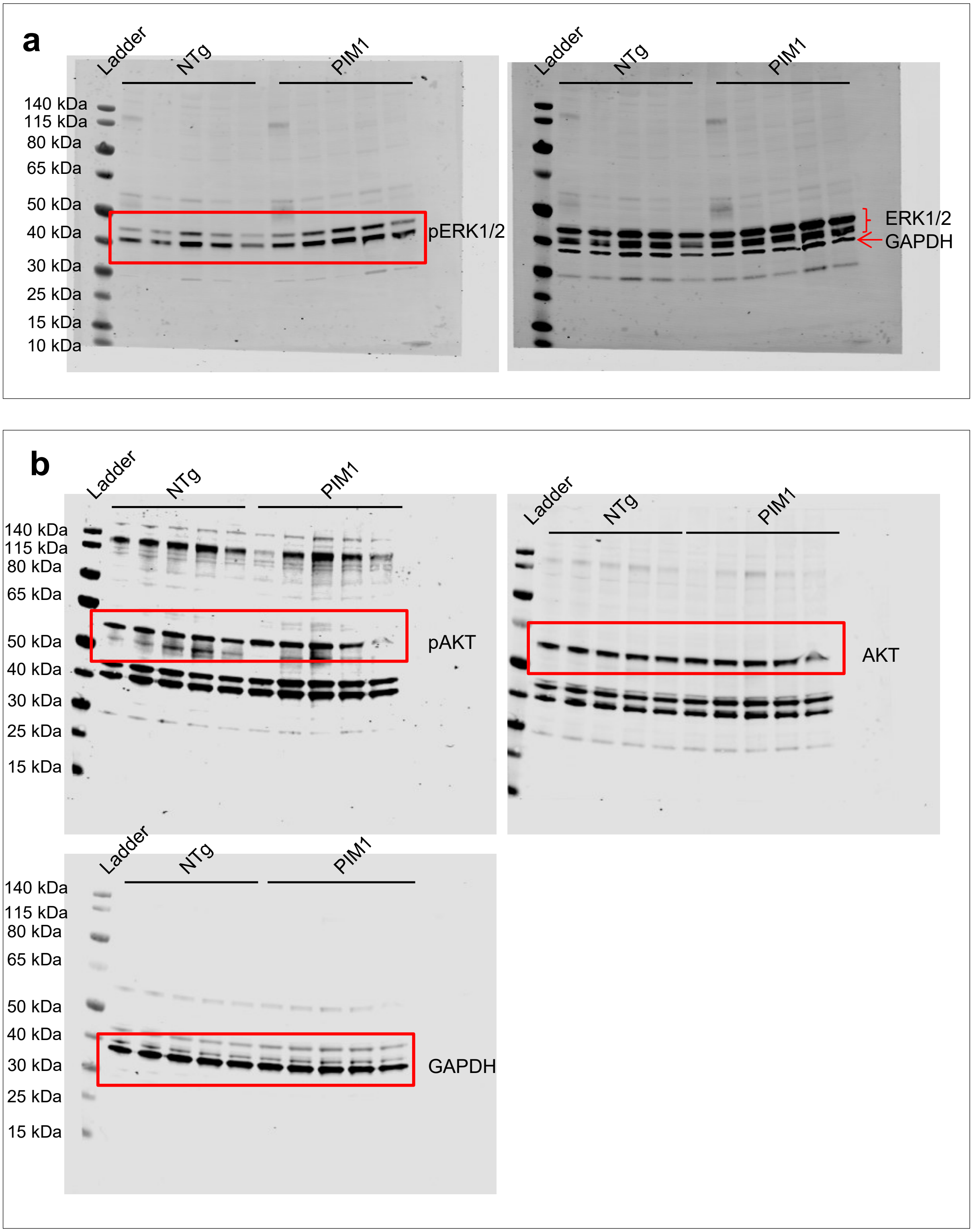


**Fig S4**: **a** Full blot of cropped image presented in Fig 3a and **b** Fig 3b of the manuscript.

Supplemental Figure 5

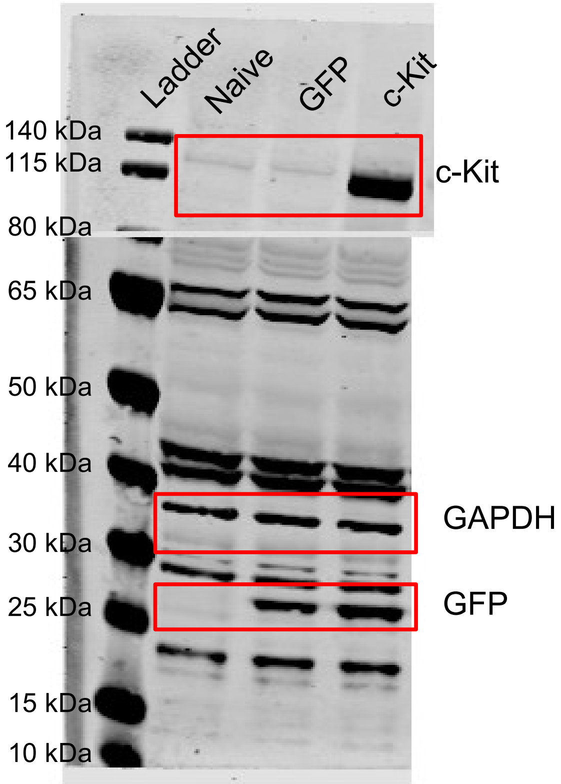


**Fig S5**: Full blot of cropped image presented in Fig 4b of the manuscript.

# Supplemental Figure 6

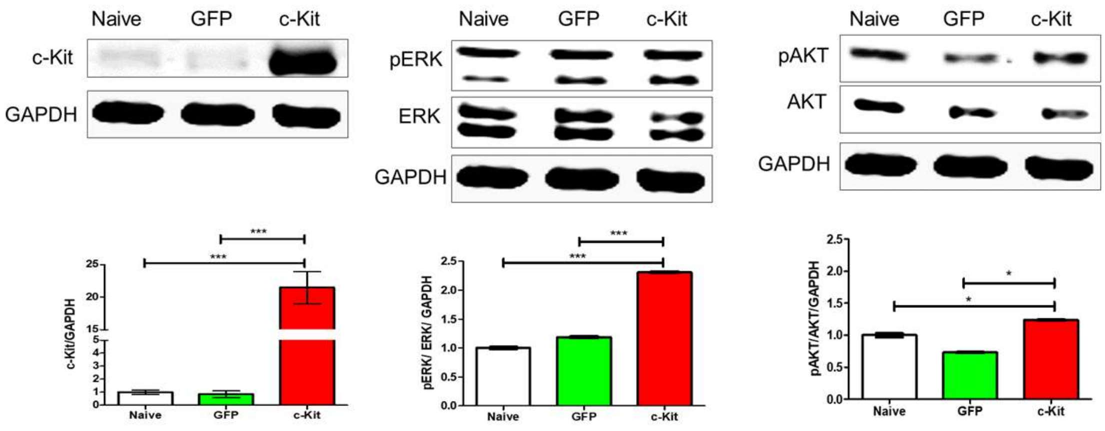


**Fig S6**: Immunoblot analysis of c-Kit, activated ERK1/2 and activated AKT in naïve and virally transduced cardiomyocytes with quantification shown below. Error bars represent SEM, *p<0.05, **p<0.01 and ***p<0.001 as measured by two-way ANOVA, multiple comparison with Tukey.

# Supplemental Figure 7

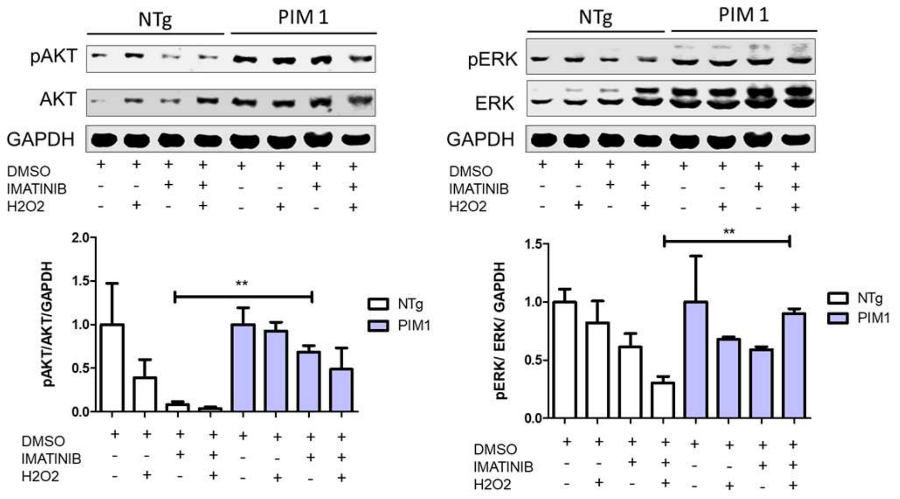


**Fig S7**: Immunoblot analysis of activated ERK1/2 and activated AKT in NTg and PIM1 overexpressing cardiomyocytes in response to oxidative stress in presence and absence of Imatinib. Quantification is shown below. Error bars represent SEM, $^{**}p<0.01$ as measured by two-way ANOVA, multiple comparison with Tukey.
